# COVID-19 vaccine hesitancy among healthcare workers and its socio-demographic determinants in Abia State, Southeastern Nigeria: a cross-sectional study

**DOI:** 10.11604/pamj.2021.40.10.29816

**Published:** 2021-09-03

**Authors:** Chidinma Ihuoma Amuzie, Franklin Odini, Kalu Ulu Kalu, Michael Izuka, Uche Nwamoh, Uloaku Emma-Ukaegbu, Grace Onyike

**Affiliations:** 1 Department of Community Medicine, Federal Medical Centre Umuahia, Abia State, Nigeria

**Keywords:** COVID-19, vaccine hesitancy, healthcare workers, pandemic, COVID-19 vaccine

## Abstract

**Introduction:**

healthcare workers are at higher risk of COVID-19 infection with ease of infection transmissibility to coworkers and patients. Vaccine hesitancy rates of 56% and up to 25% have been reported among healthcare workers in US and China respectively. Vaccination is known as the most effective strategy to combat infectious diseases. Acceptance of the COVID-19 vaccine plays a major role in combating the pandemic. This study assessed the sociodemographic factors associated with COVID-19 vaccine hesitancy among healthcare workers in Abia State.

**Methods:**

a cross-sectional study among 422 healthcare workers was conducted in Abia State with an online-based questionnaire. The questionnaire extracted information on socio-demographics and willingness to take vaccine uptake. Descriptive statistics was used to calculate frequencies and proportions. Bivariate analysis was used to test the association between the socio-demographic factors and the outcome variable (vaccine hesitancy). Logistic regression was conducted to identify the predictors of COVID-19 vaccine hesitancy. The level of significance was 5%.

**Results:**

mean age of the respondents was 40.6 ± 9.5 years and 67.1% were females The COVID-19 vaccine hesitancy rate was 50.5% (95%CI: 45.6%-55.3%). Socio-demographic factors included age, marital status, location of practice, profession, and income. Vaccine Hesitancy was predicted significantly by younger age (aOR=9.34, 95%CI:2.01-43.39), marital status (single) (aOR=4.97, 95%CI:1.46-16.97), lower income (aOR=2.84, 95%CI:1.32-6.08), and profession – Doctor (aOR=0.28, 95%CI:0.11-0.70), Nurse (aOR=0.31, 95%CI:0.15-0.64) and other allied health professionals (aOR=0.22, 95%CI:0.10-0.44).

**Conclusion:**

COVID-19 vaccine hesitancy was high among healthcare workers. Significant sociodemographic predictors influence the uptake of the COVID-19 vaccine. We recommend that the Federal and State Ministries of Health conduct awareness campaigns targeting the younger age group, singles, lower income class, and non-clinical staff.

## Introduction

The COVID-19 pandemic has resulted in so many cases and deaths all around the globe. Since the World Health Organization (WHO) was notified of an outbreak of a new disease in Wuhan, China, and its subsequent declaration as a pandemic on 11^th^ March 2020 [1], millions of cases and deaths have been reported worldwide. As of 11^th^ May 2021, a total of 160,160,122 confirmed cases with 3,326,536 deaths of confirmed COVID-19 infection have been reported globally [2]. The United States of America has had the highest number of cases worldwide, with 33,539,208 confirmed cases and 596,766 deaths [2]. As of the reference date, Nigeria had recorded 165,468 confirmed cases, 156,318 discharged and 2,065 deaths with Lagos State being the State with the highest number of cases -58,599 confirmed cases [3]. Vaccines have been one of the most successful public health interventions of all time. Over time, they have been successfully deployed in the control of vaccine-preventable diseases. Despite the wide availability of vaccines and huge success recorded in disease control, several individuals and groups still kick against vaccine use. Vaccine hesitancy (VH), as defined by the WHO refers to a delay in acceptance or refusal of vaccines despite the availability of vaccine services [4].

Healthcare workers are at a higher risk of COVID-19 infection and illness due to the ease with which infection can be transmitted to coworkers and patients [5-7]. The WHO defines healthcare workers (HCWs) as “all people engaged in actions whose primary intent is to enhance health”. This includes doctors, nurses, midwives, paramedical staff, hospital administrators and support staff, and community workers [8]. An earlier scoping review reported 3.9% of COVID-19 infections among HCWs worldwide [9]. In New York, United States, 19.4% was recorded among HCWs, [5] similar to a rate of 19% reported for healthcare personnel in the United States [10]. A rate of 10.6% was noted in Qatar and as low as 5.62% in Iran [11,12]. In Nigeria, an earlier national survey reported that 9.3% of the confirmed cases were HCWs.[13] A recent study in Southsouthern Nigeria has observed a very low rate of 2% among HCWs during the period of study [14]. Concurrently, another study in Southsouthern Nigeria noted a rate of 15.2% [15]. Healthcare workers are prioritized in almost all the countries of the world before vaccine availability [16]. However, this prioritization is not associated with optimal utilization among the HCWs [17].

A systematic review showed that the COVID-19 acceptance rate among HCWs surveyed ranged from as low as 27.7% in the Democratic Republic of Congo to as high as 78.1% in Israel [18]. A study in Nigeria recorded a vaccine hesitancy rate of 41.8% among the adult population [19]. Various determinants influence vaccine hesitancy, some of which include sociodemographic factors arising from personal interpretation of the vaccines [4]. Globally, many individual characteristics known to affect vaccine uptake have been documented in studies. Most of these factors include sex, age, education, employment, religion, income, having children at home [19-24]. Male gender is associated with vaccine hesitancy. This is very important, especially in patriarchal societies, as seen in many African countries [25]. The level of education has a major link to the receipt of information about vaccines. It is known that the less educated have poor access to information and rely on other sources for reliable information [26]. We therefore aimed in this study to assess the sociodemographic factors associated with COVID-19 vaccine hesitancy among healthcare workers in Abia State.

## Methods

**Study area and design:** this was a descriptive cross-sectional study conducted in March 2021 among healthcare workers of Abia State in Southeastern Nigeria. It had an estimated population of 3,784,355 in 2017 projected from the 2006 national population census with an annual growth rate of 3.0% [27]. Geopolitically, Abia State is divided into three senatorial zones (Abia North, Abia South, and Abia Central) with 17 local government areas and 291 political wards. The State has 517 public primary healthcare centres, 17 public secondary healthcare facilities, three public tertiary healthcare centres, and two diagnostics centres. This is complemented by many privately-owned primary healthcare facilities [27]. The study sites selected by simple random sampling included: two tertiary hospitals (Federal Medical Centre Umuahia, Abia State, and Abia State Teaching Hospital Abayi Aba, Abia State), two secondary health care facilities (General Hospital Amachara and General Hospital Ohafia), and three ward primary health care facilities (PHCs), one each from the three Senatorial Zones of the State. The ward PHCs were World Bank PHC, Eziukwu PHC and Eziama PHC.

**Study population:** Medical doctors, nurses, pharmacists, medical laboratory scientists, scientific officers, administrative officers, and other allied health professionals comprised the study population. Those eligible for the study were healthcare workers working in government-owned health facilities who had access to the internet on their smartphones and other computer devices. Eligible participants currently not working in Abia State, with debilitating illnesses that would interfere with the communication process, such as cerebrovascular accidents, and those working in COVID-19 designated isolation centres, were excluded from the study. In each of the study sites, the WhatsApp/Telegram platforms of various groups of healthcare workers were identified with the help of the heads of departments. Amid the global pandemic, members of these online platforms were recruited for the study. The main outcome of this study was COVID-19 vaccine hesitancy, while the predictor variables were the socio-demographic characteristics.

**Data collection tool and methods:** A semi-structured questionnaire created on Google forms was used to collect the data. A brief message with a link to the questionnaire was posted selectively on the WhatsApp and Telegram of different health groups within the study sites, through the admins of the groups. The questionnaire was adapted from the WHO SAGE (Strategic Advisory Group of Experts on Immunisation) vaccine hesitancy survey sample questions [28]. The introductory section of the questionnaire contained the informed consent and overview of the study. The questionnaire was structured in two different sections. The first section contained information on the socio-demographic characteristics of the participants, like age, sex, marital status, profession, educational status, income, and location of practice. In the second section, participants were asked whether they would accept receiving the COVID-19 vaccine when it became available in Abia State. The response modalities were 'yes', 'no', or 'maybe'. A pre-test with a sample size of 40 (10% of the sample size) was done in a health facility not included in the study, to improve the wording and clarity of the items on the questionnaire. The final version of the questionnaire required an approximated time of 10 minutes to be completed. Data was collected over two weeks (6^th^-20^th^ March 2021). To reduce bias introduced by self-reported data, participants were assured of the confidentiality and privacy of their responses in the introductory session of the questionnaire. To ensure that the responses were solely from the eligible participants, the first question on the questionnaire was ‘are you a healthcare worker currently working in Abia State? If 'no' was ticked, that was the end of the survey for the individual and vice versa for 'yes'. The calculated minimum sample size was 416 based on a potential vaccine hesitancy rate of 41.8% in a previous study [19], a non-response rate of 10%, a confidence level of 95%, and a 5% margin of error.

**Data analysis:** data was analyzed using the SPSS IBM version 26. Descriptive statistics was used to derive frequencies and percentages for the sociodemographic characteristics and willingness to accept the COVID-19 vaccines. The dependent outcome was the vaccine hesitancy rate with two (2) binary outcomes ('yes' and 'no'). The response options were 'yes', 'no', and 'maybe'. At the level of data analysis, 'yes' responses were recoded to '1' and 'no/maybe' were recoded to '0'. Bivariate analysis was done to compute and compare Odd Ratios (ORs) for the interpretation of the association between sociodemographic factors and vaccine hesitancy. P values less than 0.05 and confidence intervals excluding one (1) were considered significant. Multiple logistic regression was done using the vaccine hesitancy as the outcome variable and the socio-demographic factors as the predictor variables to compute the adjusted odds ratios (AORs). The level of significance was predetermined at a p-value of less than 0.05 with a 95% confidence interval.

**Ethical consideration:** ethical approval was obtained from the Health Research Ethics Committee of the Federal Medical Centre Umuahia, Abia State, Nigeria with reference number (FMC/QEH/G.596/Vol.10/497). Respondents were informed that their participation was voluntary, and consent was implied upon completion of the questionnaire.

## Results

**Sociodemographic characteristics of respondents:** The socio-demographic characteristics of the respondents are shown in table 1. The questionnaire was completed by a total of 422 respondents. The mean age of the respondents was 40.6 ± 9.5 years. The majority of the respondents were females (67.1%) and 163(38.6%) were in the 30-39 years age group. Most of the respondents were single (76.5%). The majority were practising in the Abia Central Senatorial Zone (65.4%) and were in the category of other allied health professionals (32.9%). Furthermore, the great majority were university degree holders (56.2%) with 65.4% of them earning less than NGN200,000 (approx. 526 USD) (Table 1).

**Table 1:** socio-demographic characteristics of respondents (N=422)

Variable	Frequency	Percentage (%)
**Sex**		
Male	139	32.9
Female	283	67.1
**Age group (years)**		
20-29	40	9.5
30-39	163	38.6
40-49	138	32.7
50-59	64	15.2
>59	14	3.3
**Mean(SD)**	40.6±9.6	
**Marital status**		
Single	323	76.5
Married	81	19.2
Divorced/widowed	18	4.3
**Location of practice**		
Abia central	276	65.4
Abia south	108	25.6
Abia north	38	9.0
**Profession**		
Doctor	95	22.5
Nurse	132	31.3
Other allied professions	139	32.9
Non-clinical staff	56	13.3
**Highest educational attainment**		
Fellowship/ PhD	48	11.4
Masters/membership	96	22.7
University degree	237	56.2
WAEC O level	41	9.7
**Monthly salary (Naira)**		
< 200,000	276	65.4
200,000-400,000	81	19.2
≥400,000	65	15.4

┼ n=419

**Figure 1:**
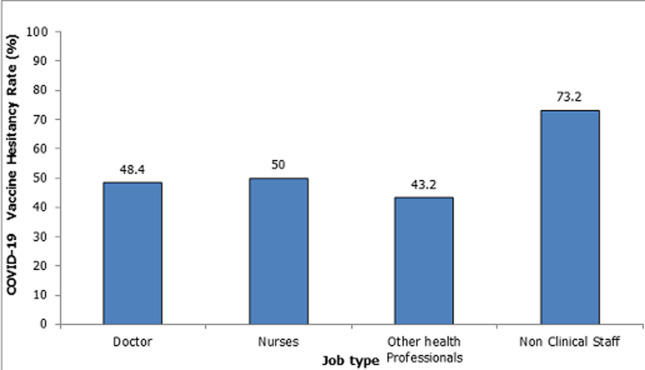
COVID-19 vaccine hesitancy rates by profession

**Prevalence of vaccine hesitancy:** the overall prevalence of Vaccine Hesitancy among the respondents was 50.5% (95%CI: 45.6%– 55.3%). Across the different professions, a higher proportion of non-clinical staff (73.2%) were more hesitant about the COVID-19 vaccine compared to other cadres of staff (Figure 1).

**Distribution of COVID-19 vaccine hesitancy rates by respondents´ characteristics:** Vaccine hesitancy was sixfold higher among those aged 20-29 years compared to those over 50years (OR = 6.59, 95%CI:1.71-25.46). Respondents who were single were thrice more likely to be hesitant compared to those who were divorced or widowed (OR = 3.40, 95% CI: 1.15-10.00). Respondents practising in the Abia South Senatorial Zone were twice more likely to be vaccine-hesitant compared to those practising in the Abia Central Senatorial Zone (OR = 2.00, 95% CI: 1.27-3.16). Participants who were doctors (OR = 0.34, 95%CI: 0.16-0.70), nurses (OR = 0.36, 95%CI: 0.18-0.72) or other allied health professionals (OR = 0.27, 95% CI:0.14-0.54) were less likely to be vaccine-hesitant compared to non-clinical staff. Being in the category of NGN200,000- NGN400,000 income group had higher odds of vaccine hesitancy compared to those within the income level of above NGN400,000 (Approx. 1051USD) (OR = 3.00, 95% CI:1.52-5.91) (Table 2).

**Table 2:** hesitancy to potential COVID-19 vaccine by sociodemographic characteristics (N=422)

Variables	Hesitancy to COVID-19 vaccine		Total (%)	OR (95% C1)	P-value
	Yes n(%)	No n(%)			
**Sex**					
Male	75(54.0)	64(46.0)	139(100)	1.23(0.82- 1.85)	0.316
Female®	138(48.8)	145(51.2)	283(100)	1	
**Age group(years)**					
20-29	29(72.5)	11(27.5)	40(100.0)	6.59(1.71- 25.46)	0.006
30-39	71(43.6)	92(56.4)	163(100.0)	1.93(0.58- 6.41)	0.283
40-49	70(50.7)	68(49.3)	138(100.0)	2.57 (0.77-8.60)	0.125
50-59	37(57.8)	27(42.2)	64(100.0)	3.43(0.97-12.09)	0.056
≥59®	4(28.6)	10(71.4)	14(100.0)	1	
**Marital status**					
Single	51(63.0)	30(37.0)	81(100.0)	3.40(1.15-10.00)	0.026
Married	156(48.3)	167(51.7)	323(100.0)	1.86(0.68-5.10)	0.222
Divorced/ widowed®	6(33.3)	12(66.7)	18(100.0)	1	
**Location of practice**					
Abia Central	124(44.9)	152(55.1)	276(100.0)	1	
Abia South	67(62.0)	41(38.0)	108(100.0)	2.00(1,27-3.16)	0.003
Abia North	22(57.9)	16(42.1)	38(100.0)	1.68(0.84-3.35)	0.136
**Profession**					
Doctor	46(48.4)	49(51.6)	95(100.0)	0.34(0.16-0.70)	0.003
Nurses	66(50.0)	66(50.0)	132(100.0)	0.36(0.18- 0.72)	0.004
Other health professionals	60(43.2)	79(56.8)	139(100.0)	0.27(0.14- 0.54)	0.001
Non-clinical staff®	41(73.2)	15(26.8)	56(100.0)	1	
**Highest educational attainment**					
Fellowship/PhD	22(45.8)	26(54.2)	48(100.0)	0.98 (0.42-2.26	0.962
Masters	48(50.0)	48(50.0)	96(100.0)	1.16 (0.56-2.41)	0.695
University degree	124(52.3)	113(47.7)	237(100.0)	1.27 (0.65-2.47	0.480
Diploma/ school certificate®	19(46.3)	22(53.7)	41(100.0)	1	
**Monthly salary (Naira)**					
< 200,000	133(48.2)	143(51.8)	276(100.0)	1.40(0.81 -2.42)	0.235
200,000-400,000	54(66.7)	27(33.3)	81(100.0)	3.00(1.52-5.91)	0.001
≥400,000®	26(40.0)	39(60.0)	65(100.0)	1	

P values less than 0.05 are considered significant**;** ® Reference category OR odds ratio PhD doctor of philosophy, CI confidence interval, ^┼^ n=419

**Sociodemographic predictors of COVID-19 vaccine hesitancy:** among the respondents’ sociodemographic characteristics, younger age (aOR = 9.34, 95% CI:2.01-43.39), Marital status (singles) (aOR = 4.97, 95% CI:1.46-16.97), profession – doctor (aOR=0.28, 95%CI:0.11-0.70), nurse (aOR=0.31, 95%CI:0.15-0.64), other allied health professionals (aOR=0.22, 95%CI:0.10-0.44) and lower income (aOR = 2.84, 95% CI:1.32-6.08) were the predictors of COVID-19 vaccine hesitancy (Table 3).

**Table 3:** sociodemographic predictors of potential COVID-19 vaccine hesitancy among Healthcare workers in Abia State (N=422)

Variables	AOR	95%CI	P value
**Age Group (years)**			
20-29	9.34	2.01-43.39	0.004
30-39	3.08	0.83-11.43	0.092
40-49	4.20	1.16-15.26	0.029
50-59	5.29	1.39-20.18	0.015
>59®	1		
**Marital status**			
Married	2.77	0.94-8.13	0.064
Single	4.97	1.46-16.97	0.010
Divorced/ widowed®	1		
**Profession**			
Doctor	0.28	0.11-0.70	0.006
Nurses	0.31	0.15-0.6	0.002
Other allied health professionals	0.22	0.10-0.44	0.001
Non-clinical staff®	1		
**Income (naira)**			
< 200,000	0.93	0.41-2.10	0.865
200,000 - 400,000	2.84	1.32-6.08	0.007
>400,000®	1		

P values less than 0.05 are considered significant**;** ®reference category CI confidence interval AOR adjusted odds ratio

## Discussion

This study aimed to assess the socio-demographic factors associated with COVID-19 vaccine hesitancy among healthcare workers of the State. We observed that half of the respondents reported COVID-19 vaccine hesitancy. The predictors for COVID-19 vaccine hesitancy included – age, marital status, income and profession. This value is similar to a previous report of 50% among HCWs from the south of the United States [29]. It is also consistent with the report from an additional study in the United States, with a VH rate of 56% among healthcare workers [24]. However, this contrasts with the reported VH rates in different countries. A systematic review of VH among healthcare workers observed a VH rate of more than 70%, as only 27.1% accepted to willingly be vaccinated in DRC, as well as 21.9% of healthcare workers accepting the vaccine in Israel [18]. Much lower rates were also recorded in Saudi Arabia (11%) [22] and in France where two studies reported rates of 16% and 28.4% [30,31]. However, this contrasts with the findings from surveys on the general population with rates of 22% in Nigeria and 16.4% in China [19,32]. This finding can be attributed to the fact that people are concerned about the distrust of vaccine safety and vaccine novelty which are documented as deterrents of vaccination [33,34]. Additionally, they have access to few published scientific facts on the efficacy and safety of the COVID-19 vaccines [24,35]. This could also be explained in part, by the lack of trust in the government regarding the response activities to the pandemic [36-39]. Another form of distrust that has been documented to hinder COVID-19 uptake is the lack of trust in the pharmaceutical industry [36]. Additionally, this can also be attributed to the inability to detect fake news in the form of conspiracy theories and unfounded rumours [40]. In light of this, interventions and policies to improve the vaccination of healthcare workers should be done. Age was a predictor of COVID-19 VH amongst HCWs in Abia State.

Healthcare workers of younger age had an increased odds of vaccine hesitancy. This finding is consistent with several studies which reported vaccine acceptance increased with increasing age [24,41-44]. However, this is in contrast with studies that observed that respondents of the older age group were less likely to accept the COVID-19 vaccine [34,45]. This is probably due to the reported lesser severity of COVID-19 amongst people of younger age and their exposure to a variety of different online anti-vaccination materials compared to the older HCWs. Additionally, HCWs of younger age in Abia State may have a lower risk perception of COVID-19 which can affect their willingness to take the COVID-19 vaccine. Workplace education of healthcare workers in this category, on the benefits of vaccination is highly opted for.

According to findings of this study, marital status was a significant predictor of COVID-19 VH amongst HCWs in Abia State. It was found that VH was fourfolds higher in single HCWs compared to those who were in different forms of relationships. This finding is similar to a study done in Saudi Arabia and China where there was a positive association between those married and vaccine acceptance [20,46]. However, this finding is inconsistent with studies done in Bangladesh and Lebanon where married people were more vaccine-hesitant compared to the singles [45,47]. Generally, single HCWs in Abia State are expected to be those in younger age groups. They are more likely to exhibit carefree attitudes compared to the married HCWs. The married HCWs are prone to be much older, with family ties, and tend to be intentional about their health. Also, as it is known that COVID-19 infection tends to be more severe in older age groups, a majority of married HCWs may fall into this older age category and will tend to understand the severity of this infection and be less hesitant towards getting immunized against the infection. Sensitization campaigns on vaccine hesitancy at the workplace with special considerations for the singles are highly emphasized. Income was one of the socio-demographic predictors of COVID-19 VH amongst HCWs in Abia State. This study found out that HCWs who earn between NGN 200,000 - NGN400,000 monthly were twice more likely to be hesitant about receiving the COVID-19 vaccine than other HCWs who earned less than NGN200,000 or more than NGN400,000 monthly. This is similar to a study in Japan among the general populace which noted that vaccine acceptance was lower among those with low income [48]. Conversely, it is consistent with the findings of the studies done in Bangladesh and the US where it was reported that households with lower income were more likely to be hesitant to get immunized against COVID-19 [23,24,45].

This can be explained by low awareness of vaccination benefits among these categories of people. Another reason could be financial concerns about getting vaccinated [36,49]. A unique finding of this study that could also relate to income is vaccine hesitancy based on profession with the non-clinical staff having the highest odds of vaccine hesitancy. Pecuniary measures such as cash rewards following vaccination, and tokens to alleviate transport costs to the vaccination sites are highly encouraged. Public health information on the negative effects of vaccine hesitancy should be made available to this category of workers.

Finally, profession was a statistically significant socio-demographic predictor of COVID-19 VH amongst HCWs in Abia State. Vaccine hesitancy was less likely to occur amongst clinical staff consisting of doctors, nurses, and other clinical health professionals compared to the non-clinical staff. Similar findings have been documented in studies where doctors reported willingness to be vaccinated compared to other medical cadres [25,44]. However, a country-level analysis observed that in Canada, Spain, and the UK, the highly educated were linked to lower acceptance of the COVID-19 vaccine [50]. The clinical staff may tend to be more receptive to getting vaccinated compared to the non-clinical staff, who is likely to have a reduced perceived susceptibility of occupational exposure as well as reduced perceived benefits of vaccination. Furthermore, those in the clinical cadre are likely to have more access to scientific sources of information regarding COVID-19 by virtue of their occupation and training, and this may also contribute to them being less hesitant about COVID-19 vaccination than the non-clinical staff, as reported in this study. A health awareness campaign towards the benefits of vaccination is highly recommended with a special focus on categories of healthcare workers in this category.

**Limitations:** one of the shortcomings of this study may have been selection bias, because only those with access to smartphones and computer devices could participate. This could also be a threat to the external validity of this study. However, the selection of study participants from the three Senatorial Zones of the State, with a random selection of study sites from the three different levels of healthcare facilities, made the study sample representative of the general population of HCWs in Abia State, Nigeria. **Strength:** the aforementioned limitations notwithstanding, the study draws its major strength from the fact that it was the first to assess COVID-19 vaccine hesitancy in the state, hence, providing baseline information for use by stakeholders and policymakers in the health sector in Abia State.

## Conclusion

In this study, 1 in 2 healthcare workers in Abia State was COVID-19 vaccine-hesitant. Age, marital status, profession and income were the significant sociodemographic predictors reported in the study. We, therefore, recommend that policymakers and stakeholders in the federal and state ministries of health should focus mainly on health education campaigns targeting the younger age group, those who are singles, non-clinical staff, and those in the lower income category to improve the willingness to accept the COVID-19 vaccine. Further research, including objective assessment of vaccine hesitancy, is also recommended.

### What is known about this topic

- Different vaccine hesitancy rates have been documented among healthcare workers in different countries across the globe;
- Several sociodemographic predictors are reported in different studies based on the context-specific concept of vaccine hesitancy.

### What this study adds

- This study is the first of its kind to measure COVID-19 vaccine hesitancy in Abia State;
- It identified a wide range of HCWs with higher odds of COVID-19 vaccine hesitancy relating to their sociodemographic characteristics in Abia State; this provides baseline evidence for future research and policy design to tackle vaccine hesitancy among HCWs.
